# A Taiwanese Propolis Derivative Induces Apoptosis through Inducing Endoplasmic Reticular Stress and Activating Transcription Factor-3 in Human Hepatoma Cells

**DOI:** 10.1155/2013/658370

**Published:** 2013-10-03

**Authors:** Fat-Moon Suk, Gi-Shih Lien, Wei-Jan Huang, Chia-Nan Chen, Shao-Yu Lu, Ying-Chen Yang, Ming-De Yan, Yu-Chih Liang

**Affiliations:** ^1^Division of Gastroenterology, Department of Internal Medicine, Wan Fang Hospital, Taipei Medical University, Taipei 116, Taiwan; ^2^Graduate Institute of Pharmacognosy, College of Medicine, Taipei Medical University, Taipei 110, Taiwan; ^3^New Drug Research and Development Center, NatureWise Biotech & Medicals Corporation, Nankang, Taipei 115, Taiwan; ^4^School of Medical Laboratory Science and Biotechnology, College of Medical Science and Technology, Taipei Medical University, 250 Wuxing Street, Taipei 11031, Taiwan; ^5^Department of Animal Science, College of Bioresources, National Ilan University, Ilan 260, Taiwan; ^6^Center of Excellence for Cancer Research, Taipei Medical University, Taipei 110, Taiwan; ^7^Traditional Herbal Medicine Research Center, Taipei Medical University Hospital, Taipei 110, Taiwan

## Abstract

Activating transcription factor-(ATF-) 3, a stress-inducible transcription factor, is rapidly upregulated under various stress conditions and plays an important role in inducing cancer cell apoptosis. NBM-TP-007-GS-002 (GS-002) is a Taiwanese propolin G (PPG) derivative. In this study, we examined the antitumor effects of GS-002 in human hepatoma Hep3B and HepG2 cells *in vitro*. First, we found that GS-002 significantly inhibited cell proliferation and induced cell apoptosis in dose-dependent manners. Several main apoptotic indicators were found in GS-002-treated cells, such as the cleaved forms of caspase-3, caspase-9, and poly(ADP-ribose) polymerase (PARP). GS-002 also induced endoplasmic reticular (ER) stress as evidenced by increases in ER stress-responsive proteins including glucose-regulated protein 78 (GRP78), growth arrest- and DNA damage-inducible gene 153 (GADD153), phosphorylated eukaryotic initiation factor 2**α** (eIF2**α**), phosphorylated protein endoplasmic-reticular-resident kinase (PERK), and ATF-3. The induction of ATF-3 expression was mediated by mitogen-activated protein kinase (MAPK) signaling pathways in GS-002-treated cells. Furthermore, we found that GS-002 induced more cell apoptosis in ATF-3-overexpressing cells. These results suggest that the induction of apoptosis by the propolis derivative, GS-002, is partially mediated through ER stress and ATF-3-dependent pathways, and GS-002 has the potential for development as an antitumor drug.

## 1. Introduction

Hepatocellular carcinoma (HCC) is the most frequent primary malignancy of the liver and accounts for as many as 1 million deaths annually worldwide [1–4]. The major risk factors include chronic hepatitis B virus (HBV) infection, chronic hepatitis C virus (HCV) infection, environmental carcinogens such as aflatoxin B1 (AFB1), alcoholic cirrhosis, and inherited genetic disorder such as hemochromatosis, Wilson's disease, and tyrosinemia. Among them, HBV, HCV, and AFB1 are responsible for approximately 80% of all HCC cases [4]. Despite rapid expansion of information obtained from researchers, the molecular mechanism of hepatocarcinogenesis and the molecular genetics of HCC remain elusive.

 In the past decade, induction of apoptosis has become the major strategy to combat cancer. However, resistance to apoptosis is considered to be a characteristic of several types of cancers. Therefore, a search for innovative strategies other than induction of apoptosis is urgently needed. Recent research demonstrated that the potential to induce apoptosis through endoplasmic reticular (ER) stresses can be a target for cancer therapy. Activating transcription factor-(ATF-) 3 is a member of the ATF/CREB family of basic-region leucine zipper-(bZIP-) type transcription factors [5] and is a highly versatile stress sensor for a wide range of conditions including hypoxia, hyponutrition, oxidative stresses, ER stresses, various genotoxic stresses [6, 7], and inflammatory reactions [8, 9]. ATF-3 is also activated by serum stimulation downstream of c-Myc [10] and is frequently overexpressed in various tumors including those of the prostate [11], breast [12], and Hodgkin's lymphomas [13]. Previous studies reported that ATF-3 was induced by treating cells with antitumorigenic compounds [14–18] and a phosphoinositide 3-kinase inhibitor [19]. On the other hand, ATF-3 is rapidly induced in cells treated with growth stimulators such as serum and growth factors [20]. ATF-3 induces DNA synthesis and expression of cyclin D1 in hepatocytes [21] and is involved in serum-induced cell proliferation as a target gene of c-myc [10]. In breast cancer, ATF-3 enhances cancer cell-initiating features [22] and is associated with activation of the canonical Wnt/*β*-catenin pathway [23].

**Figure 1:**
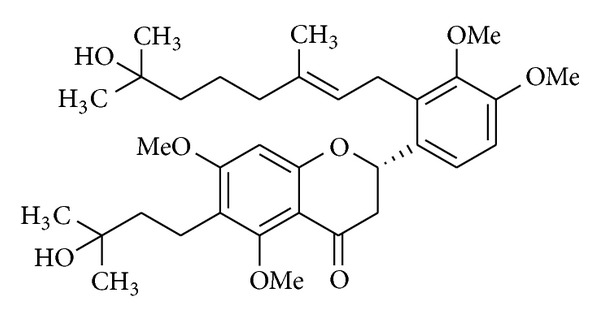
Structure of NBM-TP-007-GS-002 (GS-002).

 Besides traditional synthetic compounds, many natural products were found to exert anticancer effects. Identification of the active components and their mechanisms of action are important to assess their potential for clinical use and possible diverse side effects. Propolis, a natural resinous product, is collected from various plant sources by honeybees, which use it to seal holes in their honeycombs. Propolis was reported to exhibit a broad spectrum of activities including antibacterial, antifungal, antiviral, anti-inflammatory, antioxidant, hepatoprotective, and anticancer properties [24, 25]. Ten propolins (propolins A~J) of the active components were isolated and characterized. Our previous studies suggested that propolin G (PPG) isolated from Taiwanese green propolis induced growth inhibition and apoptosis of brain cancer cells possibly due to modulating expressions of cell cycle-regulator genes and further activating caspase cascades and mitochondrial pathways, ultimately resulting in the induction of apoptosis [26]. We were interested in developing more-potent antitumor activity from PPG and found that the PPG derivative, NBM-TP-007-GS-002 (GS-002) (Figure 1), was a more-potent antitumor drug than was the parental PPG. In this study, we further investigated the molecular mechanism of GS-002 in inhibiting hepatoma cell proliferation.

## 2. Materials and Methods

### 2.1. Materials

PPG and its derivative, GS-002 (Figure 1), were synthesized by Professor Huang (Taipei Medical University, Taipei, Taiwan), and a stock solution was made in dimethyl sulfoxide (DMSO) solvent. Inhibitors of SP600125, SB203580, and PD98059 were purchased from Tocris Bioscience (Bristol, UK). Antibodies against the cleaved poly (ADP-ribose) polymerase (PARP), Bad, phospho-p38, and p38 and a Cleavage Caspase Antibody Sampler Kit were purchased from Cell Signaling Technology (Danvers, MA). Antibodies against c-Jun N-terminal kinase (JNK), phospho-JNK, extracellular signal-regulated kinase (ERK), and phospho-ERK were purchased from BD Biosciences (San Jose, CA). An anti-*α*-tubulin antibody was purchased from Frontier Laboratories (Chicago, IL), and an anti-ATF-3 antibody was purchased from Abcam (Cambridge, MA).

### 2.2. Cell Culture and Cell Viability Assay

Human hepatoma Hep3B and HepG2 cells were purchased from the Food Industry Research and Development Institute (Hsinchu, Taiwan) and cultured in minimum essential medium (MEM) supplemented with 10% heat-inactivated fetal bovine serum (FBS), 1% nonessential amino acids, 1% sodium pyruvate, and 1% L-glutamine and maintained in a humidified incubator at 37°C with 5% CO_2_. To determine viable cells, cells were seeded in a 24-well plate at a density of 6 × 10^4^ cells/mL. Drug-treated cells were washed with phosphate-buffered saline, fixed with 2.5% glutaraldehyde, and stained with 1% crystal violet dye as described previously [27].

### 2.3. Flow Cytometric Analysis

Drug-treated cells were stained with propidium iodide (PI) (Sigma-Aldrich, St. Louis, MO) alone or double-stained with Annexin V-Alexa Fluor 488 (Life Technologies, Taiwan Brand, Taipei, Taiwan) and PI and were analyzed by FACScan flow cytometry using CellQuest 3.3 analysis software (Becton Dickinson, San Jose, CA) as described previously [28].

### 2.4. DNA Fragmentation Assay

Drug-treated cells were lysed with digestion buffer containing 0.5% sarkosyl, 0.5 mg/mL proteinase K, 50 mM Tris buffer (pH 8.0), and 10 mM EDTA at 56°C for 3 h and then treated with RNase A (0.5 *μ*g/mL) for another 2 h at 56°C. DNA was extracted with phenol/chloroform/isoamyl (25 : 24 : 1), analyzed by 1.8% agarose gel electrophoresis, stained with SYBR Green dye, visualized under UV light, and photographed.

### 2.5. Western Blot Analysis

Total cellular proteins (40 *μ*g) were resolved by 8%~12% sodium dodecyl sulfate-(SDS-) polyacrylamide gel electrophoresis (PAGE), transferred onto polyvinylidene difluoride membranes (Millipore, Bedford, MA), and visualized using enhanced chemiluminescence kits (Amersham, Arlington, IL) as described previously [28].

### 2.6. Reverse **Transcriptase-P**olymerase Chain Reaction (RT-PCR)

Total RNA was isolated from cultured cells, and complementary (c)DNA was prepared as previously described [28]. ATF-3 and glyceraldehyde-3-phosphate dehydrogenase (GAPDH) cDNAs were amplified by incubating 500 ng equivalents of total cDNA in 100 mM Tris-HCl buffer (at pH 8.3) containing 500 mM KCl, 15 mM MgCl_2_, 0.1% gelatin, 200 *μ*M of each dNTP, and 50 units/mL SuperTaq DNA Polymerase (Ambion, Austin, TX) with the following oligonucleotide primers: 5′-GCTGCAAAGTGCCGAAACAAG-3′ and 5′-TCTCCAATGGCTTCAGGGTT-3′ for ATF-3 and 5′-TGAAGGTCGGTGTGAACGGATTTGGC-3′ and 5′-CATGTAGGCCATGAGGTCCACCAC-3′ for GAPDH. Thermal cycle conditions were as follows: 1 cycle at 94°C for 5 min; followed by 25 cycles at 94°C for 30 s, 50°C for 30 s, and 72°C for 1 min; and with a final cycle at 72°C for 10 min. PCR products were analyzed on 1% agarose gels and stained with SYBR Green dye.

### 2.7. Transient Transfection

The ATF-3 overexpressing plasmid, pCI-ATF3, and ATF-3 promoter reporter plasmid, Luc-1850 (ATF-Luc-1850), were kindly provided by Professor Shigetaka Kitajima (Tokyo Medical and Dental University, Tokyo, Japan). The ATF-Luc-1850 reporter plasmid contains an 1850-bp fragment, −1850 to +50 relative to the transcription start site of the human ATF-3 gene. To overexpress ATF-3, cells were seeded in a 24-well plate at the density of 6 × 10^4^ cells/mL and transfected with the pCI-ATF3 plasmid or an empty pcDNA3 plasmid as a control using Lipofectamine 2000 (Life Technologies, Taiwan Brand).

For the ATF-3 reporter activity assay, cells were seeded in a 24-well plate at a density of 6 × 10^4^ cells/mL and transfected with the ATF-Luc-1850 reporter plasmid and phRL-TK (Promega, Madison, WI) as an internal control plasmid with Lipofectamine 2000. Total cell lysates were collected, and the luciferase activity was detected using a Dual-Luciferase Reporter Assay System (Promega) and a Plate Chameleon Multilabel plate reader (HIDEX OY, Turku, Finland) according to the manufacturer's instructions. Luciferase activities of the reported plasmid were normalized to luciferase activities of the internal control plasmid [29].

### 2.8. Statistical Analysis

Data are presented as the mean ± standard error (SE) for the indicated number of independently performed experiments. The statistical analysis was performed using one-way Student's *t*-test, and differences were considered significant at *P* < 0.05.

## 3. Results

### 3.1. GS-002 Induced Apoptosis in Human Hepatoma Cells

**Figure 2:**
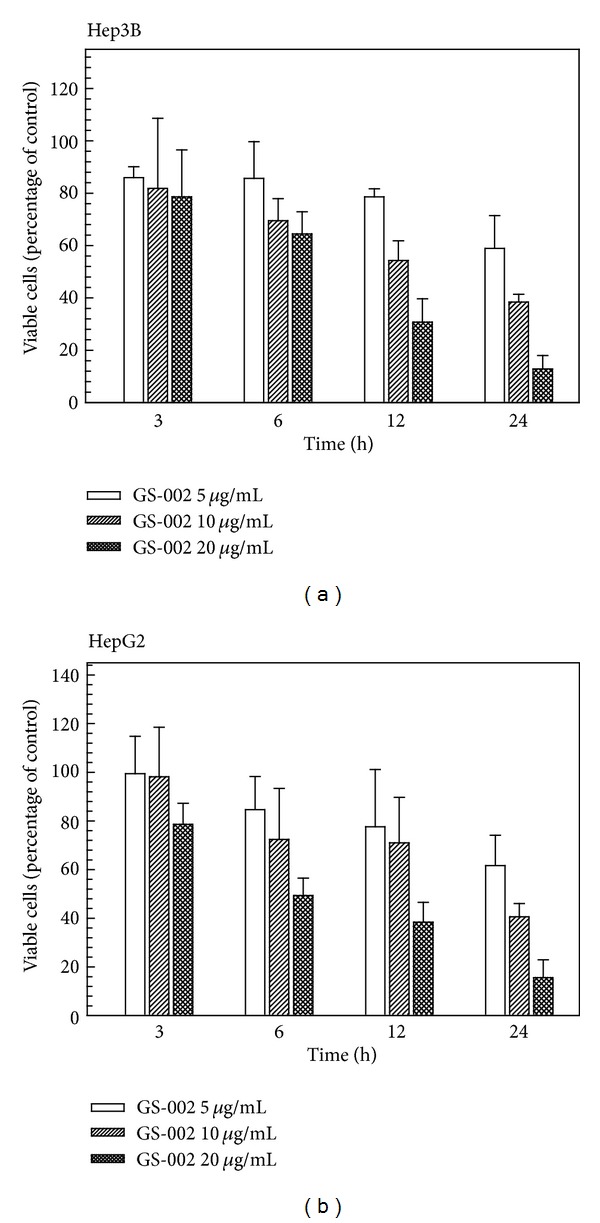
The propolis derivative, GS-002, decreased viable cell numbers in human hepatoma cells. (a) Hep3B and (b) HepG2 cells were treated with various concentrations of GS-002 for the indicated time periods, and then viable cell numbers were determined with a crystal violet dye. Values are presented as the mean ± SE of three independent experiments.

We examined the antitumor effect of GS-002 in human hepatoma cell lines and found that GS-002 significantly inhibited cell proliferation and induced cell apoptosis in dose-dependent manners (Figure 2). In the human hepatoma Hep3B and HepG2 cell lines, GS-002 significantly induced cell death in dose- and time-dependent manners and, respectively, showed 88% and 84% reductions in cell viability with 20 *μ*g/mL of GS-002 at 24 h of treatment. 

**Figure 3:**
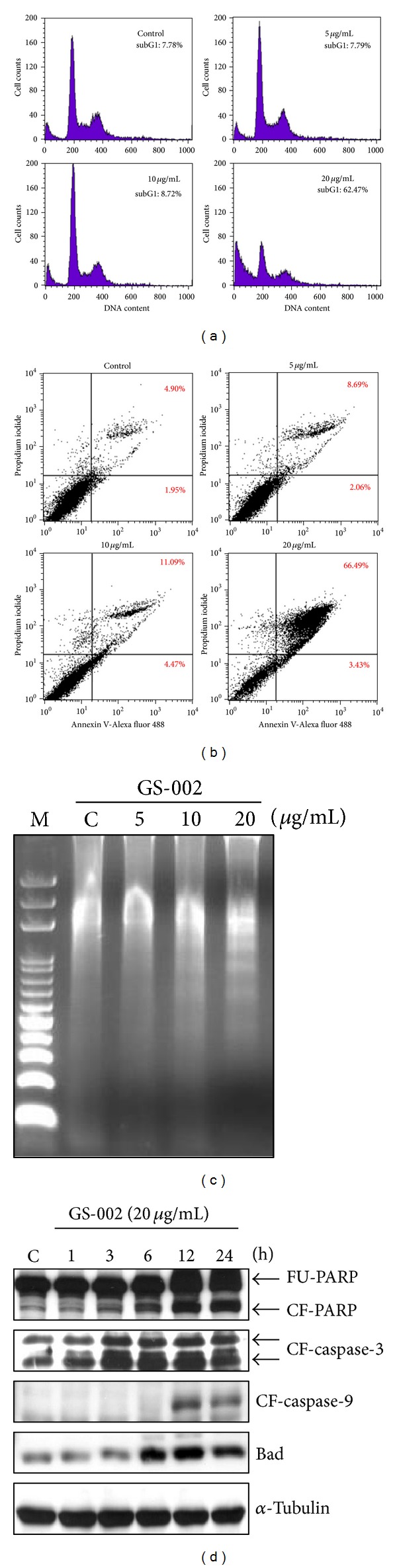
The propolis derivative, GS-002, induced cell apoptosis in human hepatoma cells. Hep3B cells were treated with various concentrations of GS-002 for 24 h, and (a) the subG1 population was determined by a flow cytometric analysis; (b) apoptotic cells were determined by a flow cytometric analysis with annexin-V/PI staining; and (c) the DNA fraction was extracted and chromatographed by agarose gel electrophoresis. (d) Hep3B cells were treated with 20 *μ*g/mL of GS-002 for the indicated time periods. Total cell lysates were used to detect the protein expression of full-length PPAR (FU-PPAR), cleaved form of PPAR (CF-PPAR), cleaved form of caspase-3 (CF-caspase-3), cleaved form of caspase-9 (CF-caspase-9), Bad, and *α*-tubulin by Western blotting.

 Cell-cycle progression was analyzed by flow cytometry with PI staining. After treatment with GS-002 for 24 h, there was no significant cell-cycle change in the G1, S, or G2/M phases compared to control cells (Figure 3(a)). However, a marked increase in the subG1 apoptotic population was seen in cells treated with 20 *μ*g/mL of GS-002. subG1 populations were 7.78% and 62.47% of control cells and cells treated with 20 *μ*g/mL of GS-002, respectively. Cell death was also characterized using flow cytometry with PI and Annexin V-Alexa Fluor 488 staining of Hep3B cells. The lower right quadrant of the FACS histogram represents early apoptotic cells, which were stained with the green fluorescent Alexa488 dye, and the upper right quadrant of the FACS histogram represents late apoptotic cells, which were stained with both the red-green fluorescence PI and Alexa488 dyes. As shown in Figure 3(b), the late apoptotic cell population increased from 4.90% to 66.49% in cells treated with 20 *μ*g/mL GS-002. We next questioned whether GS-002 induced apoptosis in Hep3B cells. After treatment of Hep3B cells with various concentrations of GS-002 for 24 h, genomic DNA from cells was subjected to agarose gel electrophoresis. DNA fragmentation ladders significantly increased as shown in Figure 3(c). We next determined the cleavage of PARP and activation of caspases in GS-002-treated cells. After treatment with GS-002 for 24 h, the cleavage of PARP and cleaved (i.e., activated) forms of caspases-3 and -9 and Bad were found in GS-002-treated cells in a dose-dependent manner (Figure 3(d)). These results suggest that GS-002 inhibited cell proliferation through activating an apoptotic pathway in human hepatoma cells.

### 3.2. ER Stress Is Involved in GS-002-Induced Apoptosis

**Figure 4:**
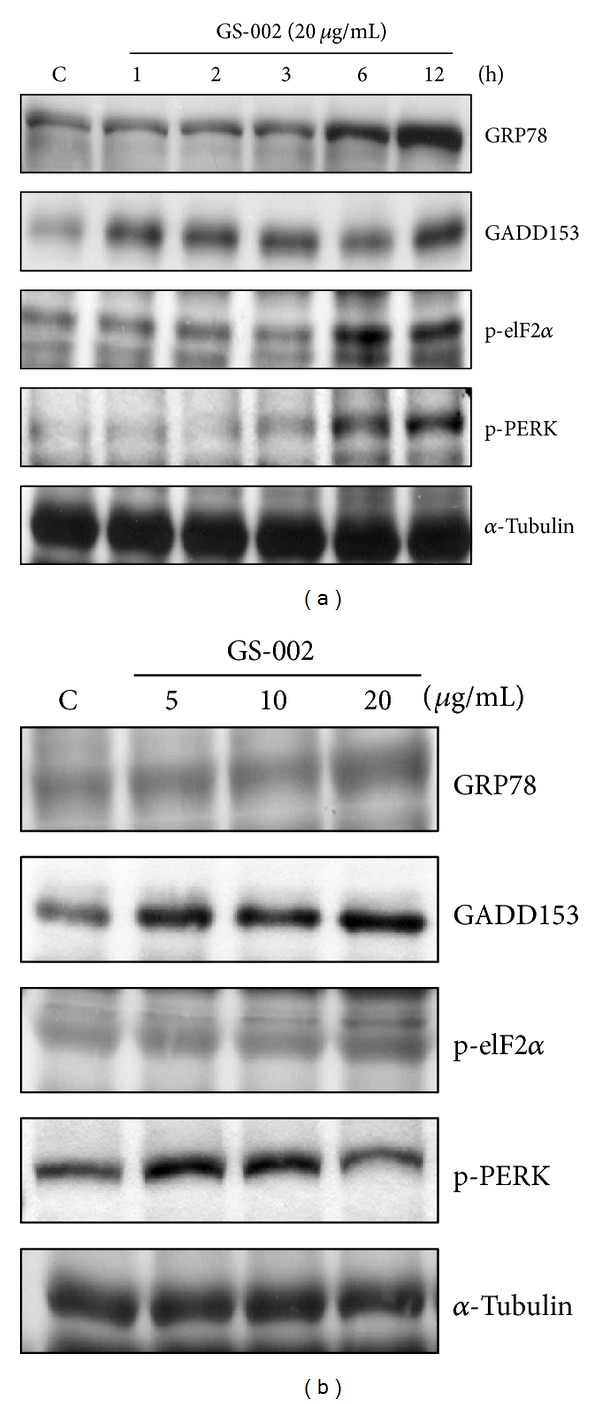
The propolis derivative, GS-002, induced endoplasmic reticular stress in human hepatoma cells. Hep3B cells were treated (a) with 20 *μ*g/mL GS-002 for the indicated time periods or (b) with various concentrations of GS-002 for 12 h. Total cell lysates were used to detect protein expressions of GRP78, GADD153, phospho-eIF2*α* (p-eIF2*α*), phosphor-PERK (p-PEK), and *α*-tubulin by Western blotting.

It was suggested that prolonged ER stress can cause cells to undergo apoptosis. To examine whether GS-002 also caused apoptosis through ER stress in human hepatoma cells, several ER-responsive proteins and ER-specific signals were detected. We first measured expressions of GRP78, which acts as a gatekeeper in activating ER stress, and GADD153, a transcription factor increased by ER stress. The Western blot analysis showed that expressions of GRP78 and GADD153 significantly increased after GS-002 treatment in dose- and time-dependent manners (Figure 4). We next detected the phosphorylation of ER-specific signals, including PERK and eIF2*α*, which are known to be activated in response to accumulation of unfolded proteins in the ER lumen. As shown in Figure 4, GS-002 indeed induced the phosphorylation of PERK and its substrate, eIF2*α*, in dose- and time-dependent manners. The results suggested that GS-002 was able to induce ER stress in Hep3B cells.

### 3.3. GS-002 Induced ATF-3 Expression through MAPK Pathways

**Figure 5:**
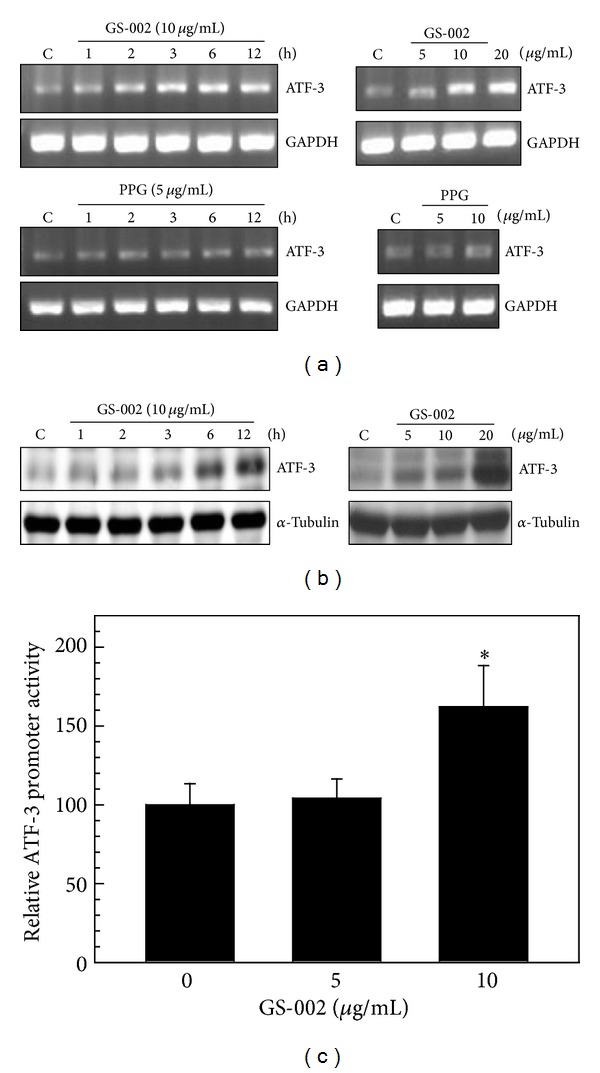
The propolis derivative, GS-002, induced ATF-3 expression in human hepatoma cells. (a) Hep3B cells were treated with various concentrations of GS-002 or PPG for 12 h (right panels), or with 10 *μ*g/mL GS-002 or 5 *μ*g/mL PPG for the indicated time periods (left panels), and total RNA was used to detect ATF-3 mRNA levels by an RT-PCR. (b) Hep3B cells were treated with various concentrations of GS-002 for 12 h (right panels) or with 10 *μ*g/mL GS-002 for the indicated time periods (left panels), and total cell lysates were used to detect ATF-3 protein levels by Western blotting. (c) Hep3B cells were transfected with 0.35 *μ*g of the ATF-Luc-1850 reporter plasmid and 0.15 *μ*g phRL-TK for 24 h and then treated with various concentrations of GS-002 for another 24 h. Total cell lysates were used to detect the luciferase activity as described in Section 2.

It is known that ATF-3 is also a stress-responsive protein, which can be induced by ER stress [30]. Next, we wanted to understand whether GS-002 can induce ATF-3 expression in human hepatoma cells. Hep3B cells were treated with GS-002, and we found that GS-002 significantly induced ATF-3 messenger (m)RNA expression in dose- and time-dependent manners (Figure 5(a)). ATF-3 protein expression also increased after GS-002 treatment (Figure 5(b)). However, the GS-002 parental compound, PPG, did not induce ATF-3 expression at a concentration of 10 *μ*g/mL (Figure 5(a), bottom). To examine whether GS-002 induced ATF-3 expression at the transcription level, we used the ATF-Luc-1850 reporter plasmid to determine the gene promoter activity of ATF-3. Hep3B cells were transfected with the ATF-Luc-1850 reporter plasmid and phRL-TK (an internal control plasmid) for 24 h and then treated with various concentrations of GS-002 for another 24 h. As for GS-002 exposure, the gene promoter of ATF-3 in Hep3B cells was upregulated in a dose-dependent manner (Figure 5(c)). These results suggest that GS-002 was able to induce ATF-3 expression at the transcription level.

**Figure 6:**
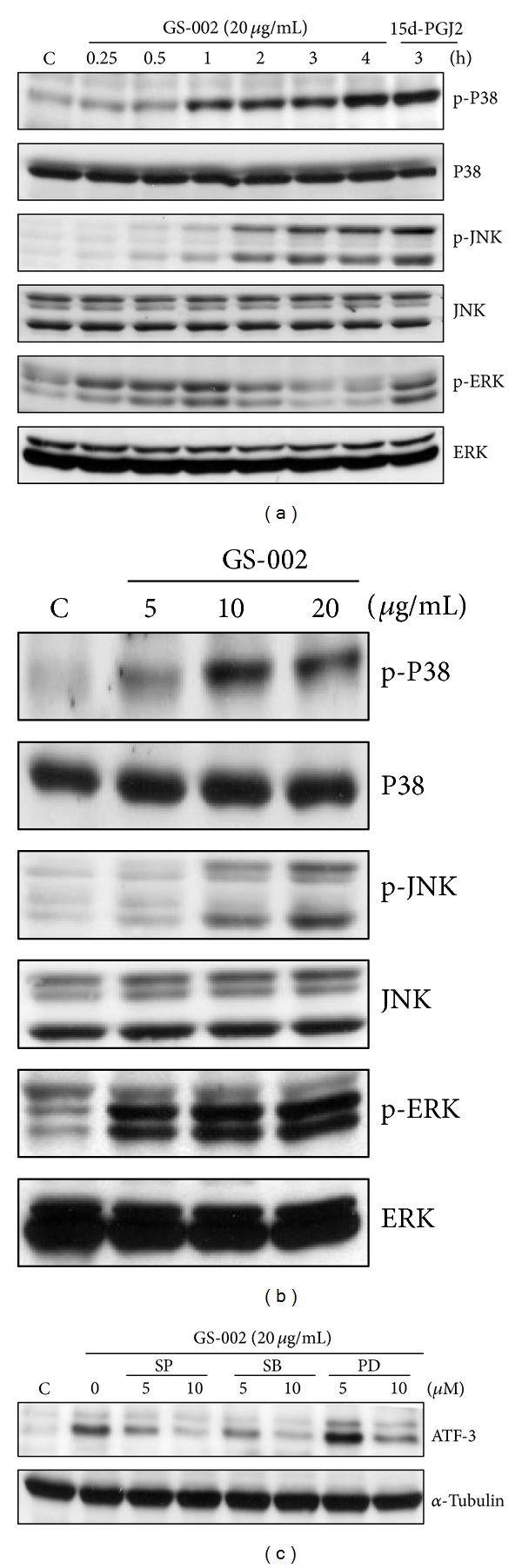
The propolis derivative, GS-002, induced ATF-3 expression that was mediated by MAPK pathways. Hep3B cells were treated with (a) 20 *μ*g/mL GS-002 for the indicated time periods, or (b) with various concentrations of GS-002 for 1 h. Total cell lysates were used to detect protein expressions of p38, phosphor-p38 (p-p38), c-Jun N-terminal kinase (JNK), phospho-JNK (p-JNK), extracellular signal-regulated kinase (ERK), and phospho-ERK (p-ERK) by Western blotting. Ten micrograms of 15-deoxy-Δ12,14-prostaglandin J_2_ (15d-PGJ_2_) was used as a positive control. (c) Hep3B cells were pretreated with 5 and 10 *μ*M of the p38 inhibitor, SB203580, the ERK inhibitor, PD98059, or the JNK inhibitor, SP600125, for 90 min, then treated with GS-002 (20 *μ*g/mL) for another 12 h, and the ATF-3 protein level was detected by Western blotting.

Activation of ATF-3 mainly depends on signaling pathways of MAPKs, which include ERK, JNK, and p38 kinase. To investigate whether GS-002 induced ATF-3 expression mediated by MAPK pathways, we examined phosphorylation levels of p38, JNK, and ERK in GS-002-treated cells. As shown in Figures 6(a) and 6(b), 20 *μ*g/mL GS-002 markedly increased phosphorylation levels of p38, JNK, and ERK. To further demonstrate the importance of the activation of p38, ERK, and JNK in ATF-3 expression in GS-002-treated cells, SB203580, PD98059, and SP600125 were used to, respectively, inhibit the activities of p38, ERK, and JNK. As shown in Figure 6(c), SB203580, PD98059, and SP600125 markedly inhibited ATF-3 protein expression of GS-002-treated cells. The results suggest that ATF-3 expression is mainly mediated by activation of MAPK pathways in GS-002-treated cells.

### 3.4. Overexpression of ATF-3 Enhanced Apoptosis in GS-002-Treated Cells

**Figure 7:**
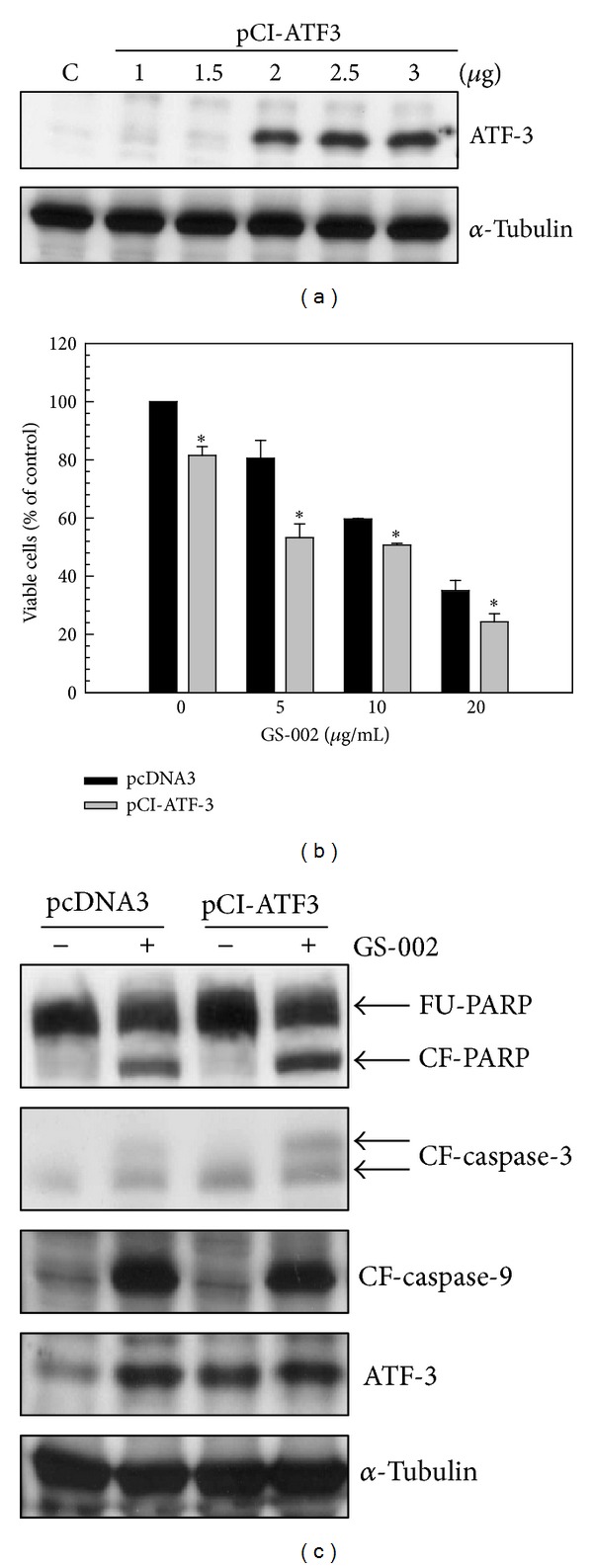
Overexpression of ATF-3 resulted in enhancement of cell apoptosis in human hepatoma cells treated with the propolis derivative, GS-002. (a) Hep3B cells were transfected with various doses of the pCI-ATF3 plasmid for 24 h, and total cell lysates were used to detect the ATF-3 protein level by Western blotting. (b) and (c) Hep3B cells were transfected with 2 *μ*g of the pCI-ATF3 plasmid for 24 h and then treated with GS-002 for another 24 h. (b) The viable cell number was determined by a crystal violet dye. Values are presented as the mean ± SE of triplcate tests. *P < 0.05 versus individual pcDNA3-transfected control cells. (c) Total cell lysates were used to detect protein expression of the full-length PPAR (FU-PPAR), cleaved form of PPAR (CF-PPAR), cleaved form of caspase-3 (CF-caspase-3), cleaved form of caspase-9 (CF-caspase-9), ATF-3, and *α*-tubulin by Western blotting.

To understand the role of ATF-3 in GS-002-induced apoptosis in hepatoma cells, Hep3B cells were transitionally transfected with the ATF-3 expression plasmid, pCI-ATF3. As shown in Figure 7(a), transfection with >2 *μ*g of pCI-ATF3 plasmid significantly increased ATF-3 protein expression. Induction of apoptosis was significantly enhanced by transfection with the pCI-ATF3 plasmid at various doses of GS-002 (Figure 7(b)), indicating that GS-002 induced greater cell apoptosis in ATF-3-overexpressing cells than in control cells. The cleaved forms of PARP and caspase-3 also increased in ATF-3-overexpressing cells (Figure 7(c)). These results suggest that induction of apoptosis by GS-002 is mediated through an ATF-3-dependent pathway.

## 4. Discussion

In this study, we demonstrated that the propolis derivative, GS-002, has the ability to inhibit cell proliferation and induce cell apoptosis in human hepatoma cells by a crystal violet assay, flow cytometry analysis, and Western blotting. GS-002 further activated ER stress and ATF-3 expression through MAPK pathways. Overexpression of ATF-3 significantly decreased cell proliferation and enhanced cell apoptosis by the transient transfection with an ATF-3 expression plasmid in GS-002-treated cells. These results therefore suggest that ATF-3 might play a key role in GS-002-induced apoptosis, and GS-002 has the potential to be developed as an antitumor drug.

 PPG was isolated from Taiwanese propolis. It was demonstrated that it could inhibit C6 glioma cell proliferation through a caspase-dependent apoptotic pathway [26]. Animal experiments indicated that PPG was able to inhibit C6 glioma cell growth in nude mice. In this study, we also found that its derivative, GS-002, had the ability to inhibit human hepatoma cell proliferation through activation of caspase cascades and the proapoptotic Bad protein. Interestingly, PPG did not induce ATF-3 expression compared to GS-002 in hepatoma cells (Figure 5(a)). This finding suggests that PPG and its derivative, GS-002, induce cell apoptosis through different signal pathways. However, GS-002 might also target other molecules which then contribute to cell apoptosis. At least, we found that ER stress increased under GS-002 treatment, but PPG did not seem to induce ER stress in hepatoma cells (unpublished data).

ATF-3 is an adaptive-response gene that regulates gene expressions to adapt to cellular microenvironmental changes [31]. Many publications indicated that ATF-3 is involved in several physiologic and pathologic processes including homeostasis, wound healing, cell adhesion, cancer-cell invasion, apoptosis, and other signal transduction pathways [32, 33]. Previous studies demonstrated that DNA damage stress, such as ionizing radiation (IR), UV radiation, and methyl methanesulphonate, can induce ATF-3 expression through different signal pathways. Overexpression of ATF-3 results in inhibition of cell proliferation, indicating that ATF-3 also plays a negative role in cell growth in response to DNA-damaging stresses [34]. Kruppel-like factor 6 (KLF6), a tumor suppressor and transcription factor, binds to the ATF-3 gene promoter and induces ATF-3 expression. However, knockdown of ATF-3 in these cells significantly blocked KLF6-induced apoptosis. Therefore, ATF3 is a key mediator of KLF6-induced apoptosis in prostate cancer cells [35]. Another experiment found that knockdown of the ATF-3 gene in mouse embryonic fibroblasts also reduced the sensitivity of cells to UV-induced apoptosis [36]. On the other hand, previous studies also found that ATF-3 has an oncogenic role. Bandyopadhyay et al. demonstrated that the tumor metastasis suppressor gene, Drg-1, inhibited the invasive ability of prostate cancer cells via downregulating expression of the ATF3 gene [37]. Moreover, another report suggested that ATF-3 has a potential dichotomous role in cancer development, since ATF3 was found to enhance apoptosis in untransformed cells but prevented apoptosis in malignant cells [12]. In this study, overexpression of ATF-3 enhanced GS-002-induced apoptosis in hepatoma cells, indicating that ATF-3 has a negative role in cell proliferation and is a key mediator in GS-002-induced apoptosis.

Regarding the inductive signal pathways of ATF-3 expression, reports indicated that ATF-3 can be induced by several different pathways. Kool et al. demonstrated that ATF-3 expression by IR was mediated by the signaling pathways of ataxia telangiectasia-mutated (ATM), Nibrin1, JNK, and p38 [38]. An experiment with UV-induced apoptosis found that p38 and JNK signaling pathways were involved in the induction of ATF-3 [39]. The induction of ATF-3 by anisomycin treatment was also mediated through a MAPK pathway in HeLa cells. p38 is a major contributor to the induction of ATF-3 compared to two other MAPK members, ERK and JNK [31]. In this study, we found that phosphorylation levels of the MAPK members, ERK, JNK, and p38, significantly increased after GS-002 treatment. Using MAPK-specific inhibitors reversed the increase in ATF-3 expression in GS-002-treated hepatoma cells (Figure 6). These results suggest that all MAPK members are important to ATF-3 induction in GS-002-treated cells. On the other hand, many studies have demonstrated that ATF-3 is a ER-stress responsive gene [40]. In this study, we demonstrated that the induction of ATF-3 might be mediated by MAPK signaling pathways in GS-002-treated cells. However, we cannot rule out the possibility that the induction of ATF-3 is mediated through ER-stress activation pathway. 

A previous report found that the induction of ATF-3 by IR in mouse thymus cells requires p53 [41]. When cells suffer injury, regardless of the state of the intracellular p53, ATF-3 is activated in a variety of cancer cells. ATF-3 expression plays a negative regulatory role in cell growth [34]. In this study, we found that GS-002 significantly induced ATF-3 expression and apoptosis in p53-null Hep3B cells, indicating that GS-002 induced ATF-3 in a p53-independent manner, and GS-002 was able to induce apoptosis in those cancer cells even with mutant p53. We cannot rule out the possibility that GS-002 can also induce ATF-3 expression through a p53-dependent pathway in other tissue types.
